# Sex differences in online ratings of healthcare professionals in Moscow: A cross-sectional study

**DOI:** 10.1016/j.pmedr.2026.103600

**Published:** 2026-08-12

**Authors:** Paul Sebo

**Affiliations:** University Institute for Primary Care (IuMFE), University of Geneva, Geneva, Switzerland

**Keywords:** Bias, Gender, Healthcare professional, Moscow, Online, Patient, Physician, Rating, Russia, Woman

## Abstract

**Objective:**

To compare online ratings between male and female healthcare professionals practicing in Moscow, Russia.

**Methods:**

This cross-sectional study used a publicly available dataset containing profiles of healthcare professionals practicing in Moscow, Russia, extracted from the Napopravku and DocDoc healthcare platforms; data extraction and analysis were performed in May 2026. Healthcare professionals with missing sex or rating data were excluded. Healthcare professions were grouped into 20 categories. Ratings were compared between men and women using the Wilcoxon rank-sum test and adjusted median regression analyses.

**Results:**

A total of 64,939 healthcare professionals were included, including 23,454 men (36.1%) and 41,485 women (63.9%). Median ratings were identical in men and women (8.6/10), although rating distributions differed significantly (*p* < 0.01). In adjusted median regression analyses, female healthcare professionals had slightly lower ratings than men after adjustment for professional experience and number of reviews (β = −0.031; *p* < 0.01). Median ratings were higher among men in 11 of 20 healthcare profession categories, whereas no category showed higher median ratings among women.

**Conclusions:**

Online ratings of healthcare professionals in Moscow were overall highly positive. Although differences were modest, male healthcare professionals tended to receive slightly more favorable ratings than female healthcare professionals across several healthcare profession categories.

## Highlights

• Online ratings were highly positive among Moscow healthcare professionals.
• Female professionals received slightly lower ratings than males.
• Men had higher median ratings in 11 of 20 healthcare profession groups.
• No healthcare profession category showed higher ratings among women.

## 1 Introduction

Online physician rating platforms have become increasingly influential in healthcare systems worldwide, potentially affecting physicians' visibility, reputation, and professional activity (Emmert et al., 2013; Hong et al., 2019; Kadry et al., 2011; Lagu et al., 2010). Multiple studies have shown that online physician ratings are generally positive (Emmert et al., 2013; Gao et al., 2012; Hong et al., 2019; Kadry et al., 2011; Lagu et al., 2010; Liu et al., 2018; Wallace et al., 2014). However, they still have important consequences, as positive and negative reviews can influence patient perceptions and healthcare decisions (Burkle and Keegan, 2015; Yaraghi et al., 2018). For example, in a survey conducted at the Mayo Clinic including 854 respondents, more than three quarters of participants reported that positive or negative online reviews could influence their decision to seek care from a physician (Burkle and Keegan, 2015).

The literature on online physician reviews has expanded considerably over the past decade. Several studies investigated factors associated with physician ratings, including physician characteristics, patient experience, and clinical outcomes (Gao et al., 2012; Kadry et al., 2011; Lagu et al., 2010; Liu et al., 2018; Lu and Rui, 2018; Ranard et al., 2016; Wallace et al., 2014; Xu et al., 2016). In addition, systematic reviews have highlighted the growing importance of physician-rating websites in healthcare decision-making (Emmert et al., 2013; Hong et al., 2019).

An emerging body of literature suggests that physician gender may influence patient evaluations and online reviews. However, most available evidence originates from the United States. In a longitudinal econometric study including 1093 physicians from Alabama, Saifee et al. found that female physicians received significantly lower online ratings than male physicians, even after adjustment for several clinical and review-related characteristics; these differences were observed across four rating domains: helpfulness, knowledge, staff, and punctuality (Saifee et al., 2022).

Similarly, Dunivin et al. analyzed 154,305 online physician reviews from Google Places in the United States and observed that reviews of female physicians were substantially more negative than reviews of male physicians (Dunivin et al., 2020). Negative reviews of female physicians more frequently referred to interpersonal characteristics such as candor, amicability, and disrespect, suggesting that female physicians may be evaluated according to different interpersonal expectations than male physicians.

More recently, in a study analyzing 345,053 written reviews of 167,150 US physicians, Madanay et al. reported that female physicians had higher odds of receiving rating penalties related to both interpersonal manner and perceived technical competence in written online reviews (Madanay et al., 2025, 2024). Other studies suggested that female physicians are more frequently evaluated according to communication style, warmth, empathy, or communal traits, whereas male physicians are more often associated with authority or competence (Chen et al., 2021; Haynes et al., 2021; Thawani et al., 2019). These observations may reflect a combination of differences in communication style and patient interaction, as well as broader social expectations and gender stereotypes in professional evaluation. Several studies have shown that physician gender may influence communication style, patient-centered behavior, consultation characteristics, and patient satisfaction (Bertakis, 2009; Hall et al., 2014; Roter et al., 2002; Schmid Mast et al., 2007). Social psychology literature further suggests that women in professional or leadership positions are frequently evaluated according to stereotypical expectations (Eagly and Karau, 2002; Heilman, 2012).

Outside the United States, evidence remains limited. In Germany, a study analyzing ratings of 23,902 dentists on the Jameda platform reported slightly more favorable ratings for female dentists than for male dentists (Emmert et al., 2015), illustrating that findings may differ across healthcare systems and sociocultural contexts. However, interpretation and comparison of studies across countries remain challenging because healthcare workforces in countries such as the United States and Germany are characterized by substantial international migration and heterogeneous cultural backgrounds, potentially influencing physician-patient interactions and patient rating behaviors (Kauff et al., 2022). According to OECD data, foreign-born physicians represented 30.2% of practicing physicians in the United States and 20.2% in Germany in 2015/2016 (Socha-Dietrich and Dumont, 2021).

To better explore healthcare professional ratings outside Western healthcare systems and in a setting characterized by lower levels of international migration, we searched for publicly available data from large emerging economies and Global South countries, including BRICS nations. Publicly available data were identified for Moscow, Russia. In contrast to the United States and Germany, the proportion of foreign migrants in the Moscow agglomeration has been estimated at approximately 9% of the total population according to recent analyses (Babkin et al., 2024). In addition, migration patterns in Moscow differ from those observed in many Western countries, as a substantial proportion of migrants originate from former Soviet republics and include populations considered culturally and linguistically closer to the local Russian population (Babkin et al., 2024). This comparatively different migration context may provide a distinct sociocultural setting in which to examine potential sex differences in healthcare professional ratings.

Using a large publicly available dataset of healthcare professionals practicing in Moscow, Russia, we aimed to compare online ratings between male and female healthcare professionals overall and across healthcare profession categories. Based on previous literature, we hypothesized that female healthcare professionals would receive slightly lower ratings than their male counterparts.

## 2 Methods

### 2.1 Study design and population

This cross-sectional study used a publicly available dataset deposited on Kaggle in 2024 entitled Healthcare Practitioners Dataset, created by Kirill Shchitaev (*“Healthcare Practitioners Dataset*”, 2024). The dataset contains profiles of healthcare professionals practicing in Moscow, Russia, and was generated through API-based data extraction from the Napopravku and DocDoc healthcare platforms. However, the dataset did not indicate from which platform (Napopravku or DocDoc) each healthcare professional profile originated, precluding platform-specific analyses. Data extraction and statistical analyses were performed in May 2026.

The original dataset included 65,060 healthcare professional profiles. Profiles with missing sex information (*n* = 82) were excluded. In addition, 39 profiles had a rating value of 0. Because it was unclear whether a value of 0 represented a true rating or the absence of a rating, these profiles were excluded from the primary analysis. The final analytical sample therefore comprised 64,939 healthcare professionals, including 23,454 men (36.1%) and 41,485 women (63.9%). In a first sensitivity analysis, these 39 profiles were alternatively considered valid observations and included in the analyses.

Healthcare professions were extracted from the “specialities” variable. Profession names originally provided in Russian were translated into English with the assistance of ChatGPT (OpenAI) and subsequently reviewed by a native Russian-speaking nurse before being manually grouped into broader healthcare profession categories to facilitate analysis. Healthcare professionals could belong to more than one healthcare profession category when multiple specialties were listed.

This study was based exclusively on a publicly available anonymized dataset and did not involve human participants. According to current Swiss law, ethical review was therefore not required.

### 2.2 Measures

Variables available in the dataset included sex, healthcare profession, experience, ratings, and number of reviews. Ratings were standardized on a 10-point scale, with 10 representing the highest possible rating. The exact methodology used to harmonize ratings across platforms, as well as the precise definition of the experience variable corresponding to years of professional experience, were not available in the dataset documentation. Information regarding healthcare professionals' age and the dates of patient reviews was also unavailable.

### 2.3 Statistical analysis

Ratings were analyzed separately according to sex. Because rating distributions were non-normal, continuous variables were summarized using medians and interquartile ranges (IQRs). Means and standard deviations were additionally reported for descriptive purposes.

Comparisons between male and female healthcare professionals were performed using the Wilcoxon rank-sum test (Mann–Whitney *U* test). Analyses were first conducted in the overall cohort. Two sensitivity analyses were then performed. First, analyses were repeated after treating the 39 rating values of 0 as valid observations rather than missing data. Second, analyses were repeated after restricting the study population to healthcare professionals with at least 10 ratings in order to evaluate the robustness of the findings among healthcare professionals with more stable rating estimates. Each healthcare professional contributed only once to the overall analyses and regression models. Assignment to multiple healthcare profession categories occurred only for the descriptive specialty-specific analyses, in which healthcare professionals with multiple listed specialties were included in each relevant category. Professional experience was additionally described according to sex and compared using the Wilcoxon rank-sum test. Associations between sex and ratings were further examined using median (quantile) regression models adjusted for professional experience, and additionally for number of reviews.

Ratings were then analyzed according to healthcare profession category. Median ratings among men and women were compared within each category, and categories were subsequently classified according to whether median ratings were higher among men, higher among women, or identical between sexes. For descriptive visualization, sex-specific median ratings were plotted across healthcare profession categories, with male and female median ratings displayed as paired points within each category.

All analyses were performed using Stata version 15 (StataCorp, College Station, TX, USA).

## 3 Results

**Fig. 1:**
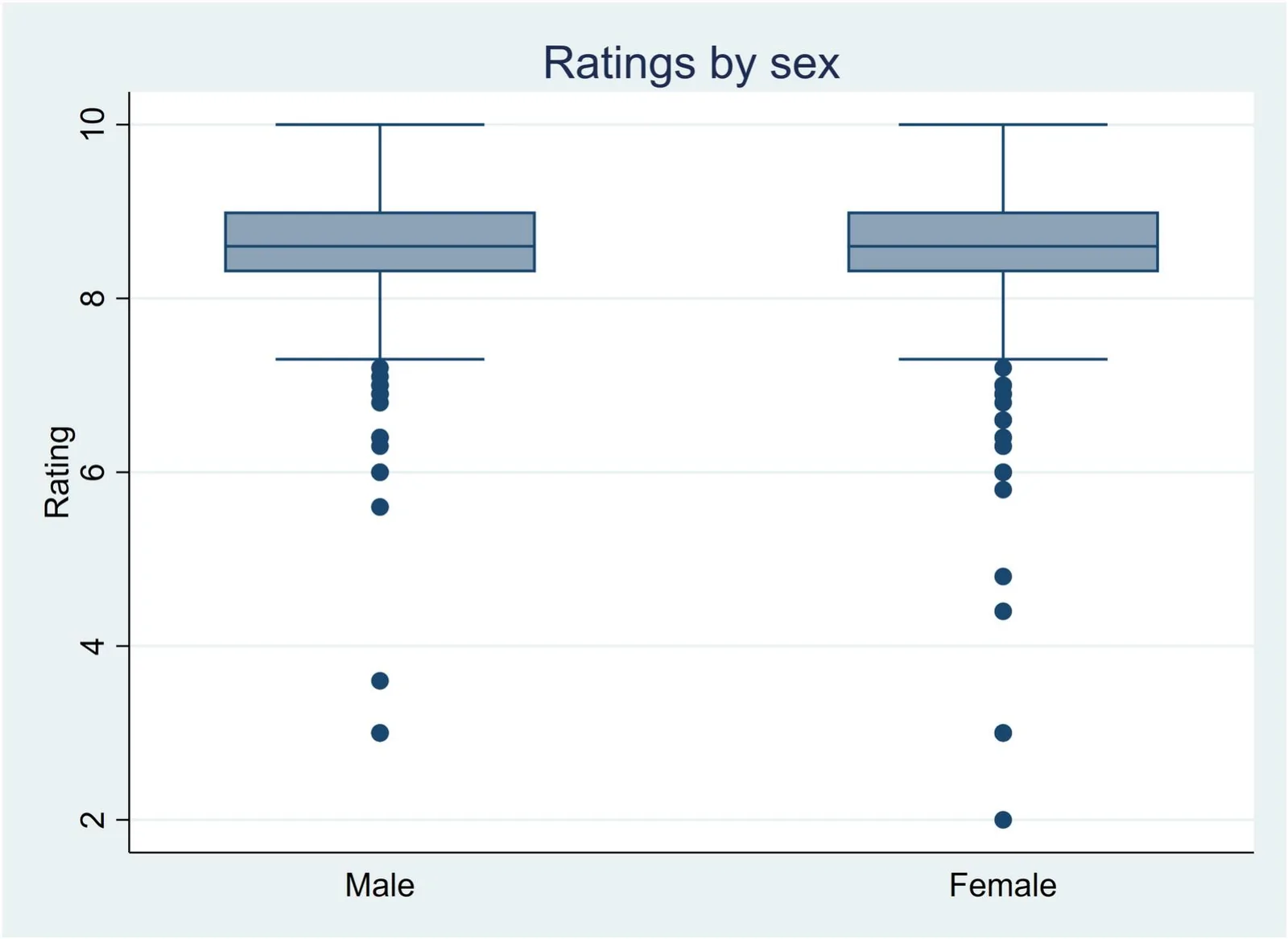
Distribution of online ratings among male and female healthcare professionals included in the study, Moscow, Russia (data extracted in May 2026).

**Fig. 2:**
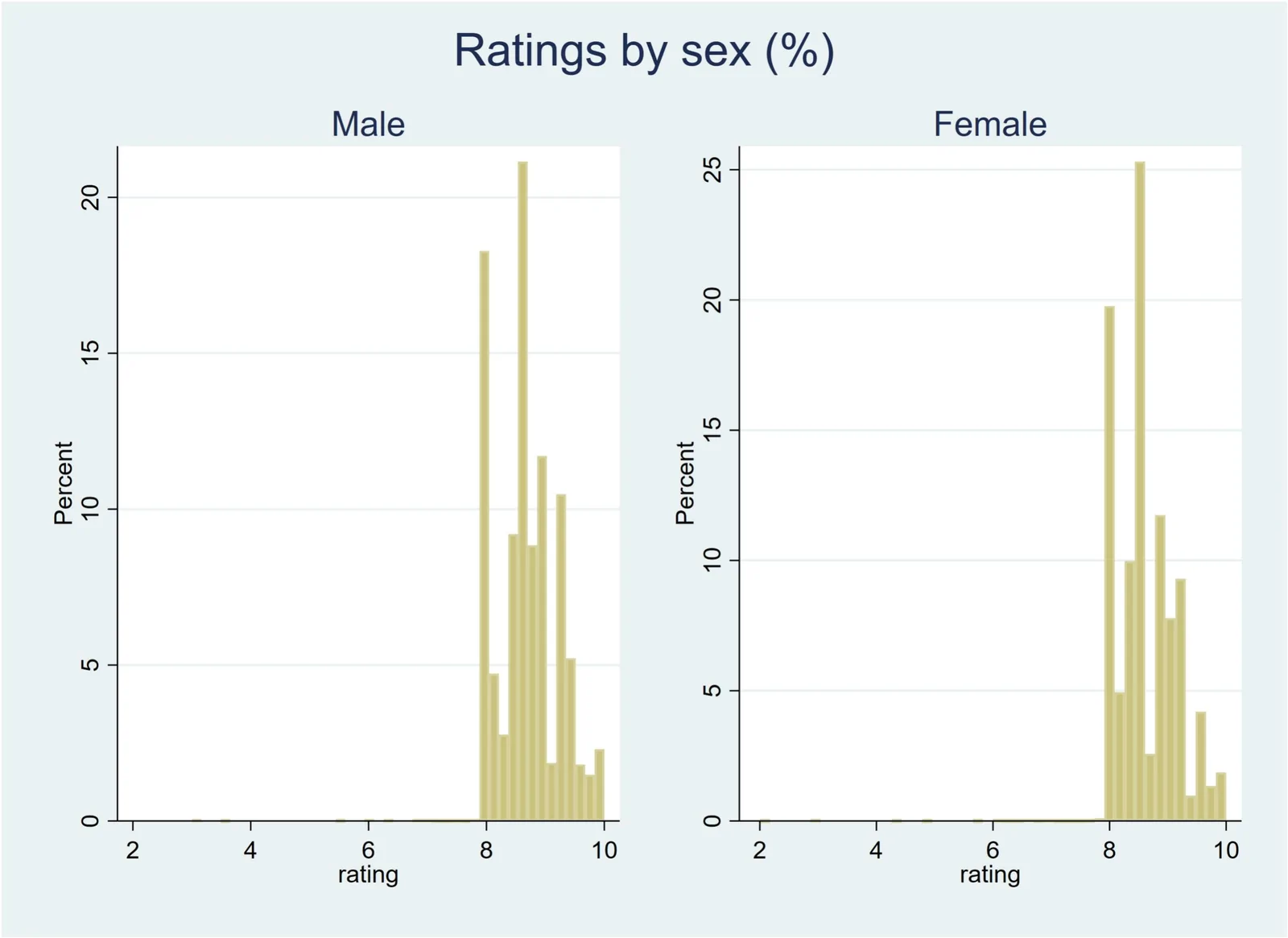
Percentage distribution of online ratings among male and female healthcare professionals included in the study, Moscow, Russia (data extracted in May 2026).

Based on data extracted in May 2026, a total of 64,939 healthcare professional profiles were included in the analysis, comprising 23,454 men (36.1%) and 41,485 women (63.9%) (Table 1). Ratings were high overall, with a median rating of 8.6/10 in both men and women. Although median ratings and interquartile ranges were similar between sexes, mean ratings were slightly higher among men than women (8.69 vs 8.65). Rating distributions differed significantly according to the Wilcoxon rank-sum test (*p* < 0.01). These findings are illustrated graphically in Fig. 1, Fig. 2. In the first sensitivity analysis, treating the 39 rating values of 0 as valid ratings rather than missing data, results were virtually unchanged. Mean ratings remained slightly higher among men than women (8.68 vs 8.64), and rating distributions continued to differ significantly according to the Wilcoxon rank-sum test (*p* < 0.01).

**Table 1:** Online ratings of male and female healthcare professionals included in the study, Moscow, Russia (data extracted in May 2026).

	n (%)	Mean (SD)	Median (IQR)	Min-max	p-value^1^
Overall cohort					<0.01
Men	23,454 (36.1)	8.69 (0.53)	8.6 (0.7)	3–10	
Female	41,485 (63.9)	8.65 (0.50)	8.6 (0.7)	2–10	
Total	64,939 (100)	8.66 (0.51)	8.6 (0.7)	2–10	
Healthcare professionals with ≥10 ratings					<0.01
Men	2422 (37.9)	9.35 (0.53)	9.4 (0.8)	7.4–10	
Female	3976 (62.1)	9.31 (0.52)	9.4 (0.9)	7.6–10	
Total	6398 (100)	9.33 (0.52)	9.4 (0.9)	7.4–10	

1 p-values were calculated using the Wilcoxon rank-sum (Mann–Whitney U) test.

In the second sensitivity analysis, restricted to healthcare professionals with at least 10 ratings, 2422 were men (37.9%) and 3976 were women (62.1%). Median ratings were again similar between sexes (9.4 in both groups), although mean ratings remained slightly higher among men (9.35 vs 9.31) (Table 1). A statistically significant difference in rating distributions persisted (p < 0.01). Ratings in this subgroup were generally higher and less dispersed than in the overall cohort.

Professional experience data were available for 57,128 healthcare professionals (median 21 years [IQR: 18; range: 1–65]). Median professional experience was slightly higher among women than men (21 years [IQR: 18; range: 1–65] vs 20 years [IQR: 17; range: 1–63], *p* = 0.02).

In median regression analyses adjusted for professional experience, female healthcare professionals had slightly lower ratings than male healthcare professionals in the overall cohort (β = −0.037, p < 0.01). This association remained statistically significant after additional adjustment for number of reviews (β = −0.031, p < 0.01).

Healthcare professions were grouped into 20 healthcare profession categories comprising 114 healthcare professions (Supplementary Table SM1). The distribution of men and women across healthcare profession categories is presented in Supplementary Table SM2. The overall total across categories was 100,538 because healthcare professionals could be assigned to more than one healthcare profession category. Marked sex differences were observed across categories. Women represented the majority of healthcare professionals in all categories except anesthesia, intensive care & emergency medicine (60.3% men), oncology (56.6% men), surgery (78.8% men), and urology & andrology (84.6% men).

**Fig. 3:**
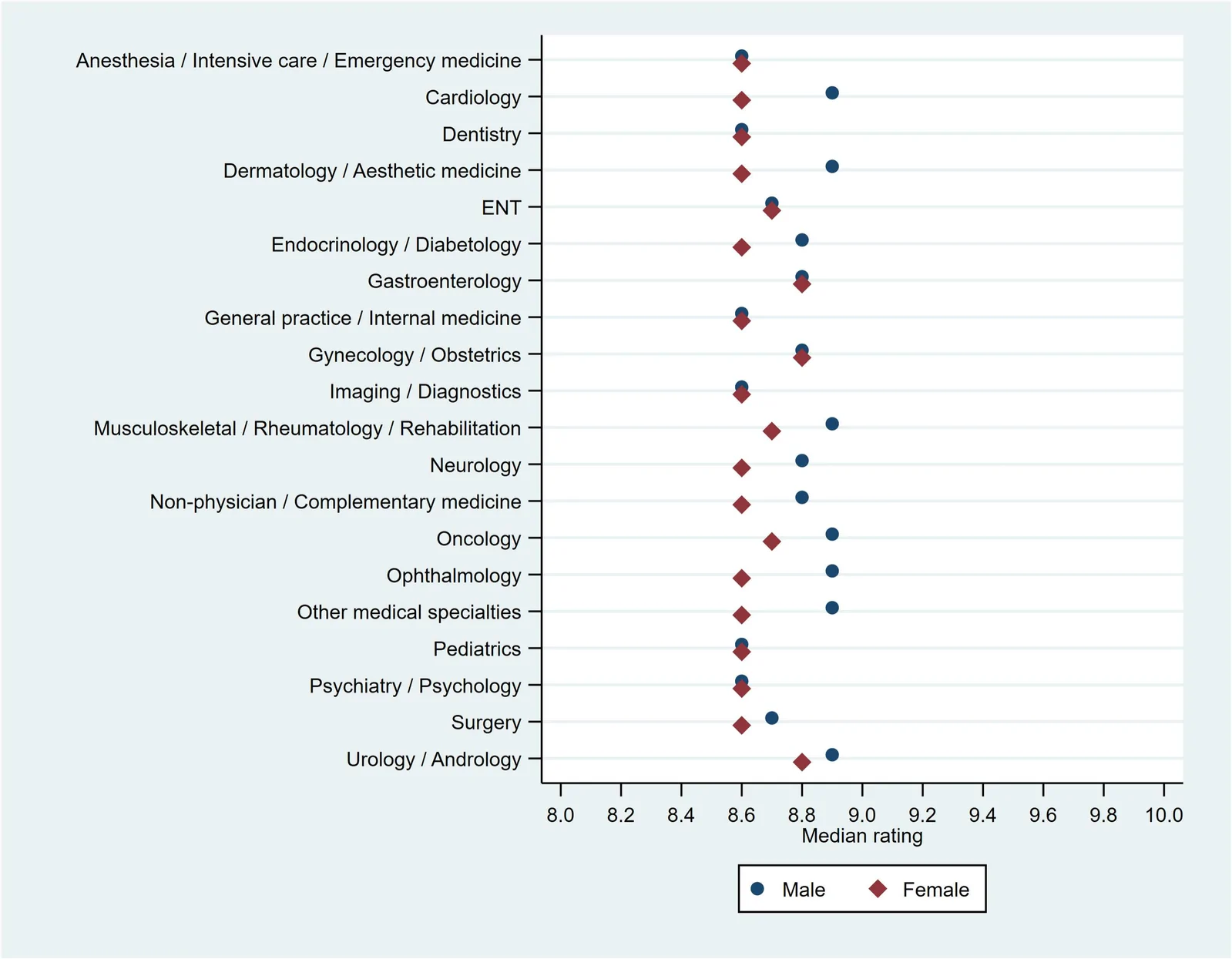
Median online ratings of male and female healthcare professionals according to healthcare profession category, Moscow, Russia (data extracted in May 2026).

Descriptive statistics of ratings according to sex across healthcare profession categories are presented in Supplementary Table SM3. Median ratings among male and female healthcare professionals across these 20 healthcare profession categories are displayed graphically in Fig. 3. Median ratings were higher among men in 11 of the 20 healthcare profession categories, whereas median ratings were identical between sexes in the remaining 9 categories. No category showed a higher median rating among women. Categories with higher median ratings among men included cardiology (8.9 vs 8.6), dermatology & aesthetic medicine (8.9 vs 8.6), endocrinology & diabetology (8.8 vs 8.6), musculoskeletal, rheumatology & rehabilitation (8.9 vs 8.7), neurology (8.8 vs 8.6), non-physician & complementary medicine (8.8 vs 8.6), oncology (8.9 vs 8.7), ophthalmology (8.9 vs 8.6), other medical specialties (8.9 vs 8.6), surgery (8.7 vs 8.6), and urology & andrology (8.9 vs 8.8). In general practice & internal medicine, median ratings were identical between men and women (8.6 in both groups).

## 4 Discussion

In this large cross-sectional study including 64,939 healthcare professionals practicing in Moscow, Russia, women represented nearly two thirds of the study population (41,485; 63.9%). Online ratings were overall highly positive, with identical median ratings in men and women (8.6/10). Nevertheless, rating distributions differed significantly according to sex, with slightly higher ratings observed among men. These differences persisted after adjustment for professional experience and number of reviews.

Marked sex differences were also observed across healthcare profession categories. Median ratings were higher among men in 11 of the 20 healthcare profession categories analyzed, whereas median ratings were identical between sexes in the remaining 9 categories. No category showed higher median ratings among women.

Our findings are generally consistent with previous studies conducted in the United States reporting slightly less favorable online ratings for female physicians (Dunivin et al., 2020; Madanay et al., 2025; Saifee et al., 2022). Saifee et al. observed lower physician ratings among women after adjustment for several clinical and review-related variables (Saifee et al., 2022), while Dunivin et al. reported more negative reviews and different interpersonal expectations toward female physicians (Dunivin et al., 2020). Similarly, Madanay et al. demonstrated that female physicians were more likely to receive penalties related not only to interpersonal behavior but also to perceived technical competence (Madanay et al., 2025).

The present study extends previous literature by examining healthcare professional ratings in Moscow, Russia, a setting characterized by substantially lower levels of international migration than those reported in the United States or Germany (Babkin et al., 2024; Socha-Dietrich and Dumont, 2021). Although healthcare systems, online review platforms, and sociocultural contexts differ substantially across countries, the persistence of slightly lower ratings among women in the present study suggests that sex-related differences in online healthcare evaluations may not be restricted to Western healthcare systems alone.

However, the magnitude of the observed differences remained small. Median ratings were identical between men and women overall and in nearly half of healthcare profession categories. These findings suggest that although statistically detectable, sex-related differences in online ratings may have limited practical magnitude at the individual level. Interestingly, no healthcare profession category demonstrated higher median ratings among women. Categories showing higher ratings among men included several highly specialized or procedure-oriented fields such as cardiology, oncology, ophthalmology, surgery, and urology & andrology. These findings suggest that perceptions of competence, authority, or technical expertise may differ according to physician sex and specialty context. They may also be interpreted through the framework of Role Congruity Theory (Eagly and Karau, 2002), which proposes that women may be evaluated less favorably when occupying roles traditionally associated with stereotypically masculine attributes such as authority, decisiveness, and technical expertise. In highly specialized or procedure-oriented fields, patients may implicitly associate technical competence and authority with male physicians. Conversely, female physicians may face a double standard in which they are expected to simultaneously demonstrate high levels of technical competence and traditionally communal characteristics such as warmth and empathy. Although our study cannot directly evaluate these mechanisms, this theoretical framework offers one possible explanation for the observed specialty-specific findings.

An additional notable finding was the marked predominance of women among healthcare professionals in the present dataset, with women representing nearly two thirds of all included healthcare professionals. This observation is consistent with previous literature reporting substantial feminization of the medical and healthcare workforce in Russia (Harden, 2001; Ramakrishnan et al., 2014). Historical, social, and economic factors have been proposed to contribute to this pattern, including the long-standing integration of women into the healthcare workforce during the Soviet period. Nevertheless, important differences persisted across specialties, with men remaining overrepresented in several surgical and procedure-oriented fields.

The increasing use of physician-rating websites in healthcare decision-making raises important questions regarding fairness and potential biases in online evaluations. Our findings suggest that sex-related differences in online ratings may persist even after accounting for professional experience and number of reviews. Although the magnitude of these differences was modest, the consistency of findings across multiple healthcare profession categories warrants further investigation into potential mechanisms underlying these disparities.

Future studies should examine additional factors potentially influencing online healthcare ratings, including physician age, communication style, consultation duration, healthcare setting, patient demographics, and written review content. These studies should also evaluate the potential contribution of physician seniority, leadership positions, and administrative responsibilities. Qualitative analyses and natural language processing approaches may further help identify whether women and men are evaluated according to different interpersonal or professional expectations.

Comparative international studies would also be valuable to better understand the influence of healthcare systems, cultural norms, and migration patterns on online physician evaluations. In particular, analyses from non-Western countries and emerging economies remain limited despite the increasing global importance of online healthcare platforms.

Several limitations should be acknowledged. First, the study relied on publicly available online platform data, and the accuracy of profile information could not be independently verified. The exact methodology used to harmonize ratings across platforms and the exact definition of the “experience” variable were not available in the dataset documentation. Furthermore, Napopravku and DocDoc may differ in user populations, review moderation policies, and rating behavior. Because the original dataset did not retain the source platform for individual profiles, we were unable to assess or adjust for potential platform-specific differences. Second, the dataset did not include several potentially important variables such as healthcare professionals' age, ethnicity, workplace characteristics, consultation volume, timing of patient reviews, or patient-level characteristics. In addition, information on professional rank, academic qualifications, or administrative responsibilities (e.g., department head or chief physician) was unavailable or incomplete, preventing adjustment for these characteristics, which may influence online ratings and partly explain the observed sex differences. Residual confounding therefore remains possible. Third, healthcare professionals could belong to multiple healthcare profession categories, which limited the possibility of performing fully independent specialty-adjusted analyses. Consequently, specialty-specific analyses should be interpreted as descriptive rather than based on mutually exclusive groups. Nevertheless, descriptive analyses across healthcare profession categories were broadly consistent with the overall statistical analyses, with higher median ratings observed among men in 11 of the 20 healthcare profession categories and no category showing higher median ratings among women. Finally, because the study was restricted to healthcare professionals practicing in Moscow, findings may not be generalizable to all healthcare professionals in Russia or other countries.

## 5 Conclusions

In this large cross-sectional study of healthcare professionals practicing in Moscow, Russia, online ratings were overall highly positive among both men and women. Although median ratings were identical between sexes, rating distributions were slightly more favorable toward men, and these differences persisted after adjustment for professional experience and number of reviews. Similar patterns were observed across several healthcare profession categories.

These findings are consistent with previous studies conducted primarily in the United States and suggest that sex-related differences in online healthcare professional evaluations may also be present in other sociocultural settings. However, the magnitude of the observed differences remained modest, and their practical implications require further investigation.

Future studies should further explore the mechanisms underlying these differences, including the potential roles of implicit bias, communication expectations, healthcare profession characteristics, and cultural context.

## Declaration of generative AI and AI-assisted technologies in the manuscript preparation process

During the preparation of this work, the author used ChatGPT (OpenAI) to improve language, grammar, and readability, and to assist with the drafting and revision of the manuscript. After using this tool, the author reviewed and edited the content as needed and takes full responsibility for the content of the published article.

## Studies in human

This study was performed in compliance with relevant laws, regulatory frameworks and guidelines where the research took place.

Ethics committee approval was not required under relevant laws and institutional guidelines. This study was based exclusively on a publicly available anonymized dataset and did not involve direct interaction with human participants. Therefore, according to current Swiss law, ethical review and approval were not required.

## CRediT authorship contribution statement

**Paul Sebo:** Writing – original draft, Methodology, Formal analysis, Conceptualization.

## Informed consent and patient details

The author has NOT obtained informed consent from participants or their legal representatives. This study was based exclusively on a publicly available anonymized dataset and did not involve direct interaction with human participants.

## Ethical approval

This study did not require ethical review, according to current Swiss law.

## Funding

The author received no specific funding for this work.

## Declaration of competing interest

The author declares that he has no known competing financial interests or personal relationships that could have appeared to influence the work reported in this paper.

## Data Availability

The data used in this study are publicly available from the Kaggle platform (Healthcare Practitioners Dataset, Kirill Shchitaev).

## Appendix A Supplementary data

Supplementary material Additional tables on healthcare profession categories and online ratings
