# Supplementary material for: Sex differences in online ratings of healthcare professionals in Moscow: A cross-sectional study
Source: Prev Med Rep. 2026 Aug 12;70:103600. doi: 10.1016/j.pmedr.2026.103600 (PMC13503096; doi:10.1016/j.pmedr.2026.103600)
Supplement: Supplementary file 1 [file mmc1.docx]

**Supplementary Material**

Supplementary Material Table SM1. Classification and frequency of healthcare professions included in the study, with 114 healthcare professions grouped into 20 categories

| Healthcare profession category | Healthcare profession | Count |
| --- | --- | --- |
| Anesthesia / Intensive care / Emergency medicine | Emergency physician | 210 |
| Anesthesia / Intensive care / Emergency medicine | Anesthesiologist-intensivist | 1442 |
| Anesthesia / Intensive care / Emergency medicine | Intensivist | 2057 |
| Anesthesia / Intensive care / Emergency medicine | Anesthesiologist | 2173 |
| Cardiology | Cardiac electrophysiologist | 64 |
| Cardiology | Cardiologist | 2357 |
| Dentistry | Dentist (gnathologist) | 114 |
| Dentistry | Dentist (periodontist) | 433 |
| Dentistry | Dentist (dental hygienist) | 545 |
| Dentistry | Dentist (implantologist) | 1142 |
| Dentistry | Dentist (orthodontist) | 1228 |
| Dentistry | Dentist (oral surgeon) | 2331 |
| Dentistry | Dentist (prosthodontist) | 2788 |
| Dentistry | Dentist (conservative dentistry) | 3512 |
| Dentistry | Dentist | 10397 |
| Dermatology / Aesthetic medicine | Mycologist | 85 |
| Dermatology / Aesthetic medicine | Trichologist | 445 |
| Dermatology / Aesthetic medicine | Dermatovenereologist | 865 |
| Dermatology / Aesthetic medicine | Venereologist | 1038 |
| Dermatology / Aesthetic medicine | Cosmetologist (aesthetic medicine) | 1894 |
| Dermatology / Aesthetic medicine | Dermatologist | 2250 |
| ENT | Phoniatrician | 24 |
| ENT | Neuro-otologist | 28 |
| ENT | Audiologist (hearing specialist) | 73 |
| ENT | ENT specialist (otolaryngologist) | 1820 |
| Endocrinology / Diabetology | Diabetologist | 63 |
| Endocrinology / Diabetology | Endocrinologist | 1571 |
| Gastroenterology | Hepatologist | 120 |
| Gastroenterology | Endoscopist | 705 |
| Gastroenterology | Gastroenterologist | 1204 |
| General practice / Internal medicine | Family physician (general practitioner) | 328 |
| General practice / Internal medicine | General practitioner (internist) | 4416 |
| Gynecology / Obstetrics | Embryologist | 106 |
| Gynecology / Obstetrics | Reproductive specialist | 338 |
| Gynecology / Obstetrics | Breast specialist | 434 |
| Gynecology / Obstetrics | Gynecologist (reproductive endocrinology) | 490 |
| Gynecology / Obstetrics | Obstetrician | 3117 |
| Gynecology / Obstetrics | Gynecologist | 4835 |
| Imaging / Diagnostics | MRI physician | 97 |
| Imaging / Diagnostics | Radiologist (imaging specialist) | 174 |
| Imaging / Diagnostics | Functional diagnostics physician | 1199 |
| Imaging / Diagnostics | Radiologist | 1413 |
| Imaging / Diagnostics | Ultrasound specialist | 3576 |
| Musculoskeletal / Rheumatology / Rehabilitation | Arthrologist (joint specialist) | 101 |
| Musculoskeletal / Rheumatology / Rehabilitation | Sports physician | 137 |
| Musculoskeletal / Rheumatology / Rehabilitation | Rehabilitation specialist | 266 |
| Musculoskeletal / Rheumatology / Rehabilitation | Vertebrologist (spine specialist) | 296 |
| Musculoskeletal / Rheumatology / Rehabilitation | Physical therapy physician | 413 |
| Musculoskeletal / Rheumatology / Rehabilitation | Rheumatologist | 417 |
| Neurology | Neurophysiologist | 30 |
| Neurology | Epileptologist | 89 |
| Neurology | Neurologist | 3270 |
| Non-physician / Complementary medicine | Vegetologist | 64 |
| Non-physician / Complementary medicine | Kinesiologist | 84 |
| Non-physician / Complementary medicine | Podiatrist | 97 |
| Non-physician / Complementary medicine | Special education specialist | 97 |
| Non-physician / Complementary medicine | Hirudotherapist | 122 |
| Non-physician / Complementary medicine | Dietitian | 306 |
| Non-physician / Complementary medicine | Speech therapist | 367 |
| Non-physician / Complementary medicine | Osteopath | 461 |
| Non-physician / Complementary medicine | Reflexologist | 631 |
| Non-physician / Complementary medicine | Physiotherapist | 737 |
| Non-physician / Complementary medicine | Manual therapist | 833 |
| Non-physician / Complementary medicine | Massage therapist | 1144 |
| Oncology | Dermato-oncologist | 58 |
| Oncology | Urologic oncologist | 59 |
| Oncology | Gynecologic oncologist | 78 |
| Oncology | Chemotherapist | 95 |
| Oncology | Breast oncologist | 184 |
| Oncology | Oncologist | 1287 |
| Ophthalmology | Ophthalmic surgeon | 206 |
| Ophthalmology | Ophthalmologist | 1707 |
| Other medical specialties | Parasitologist | 14 |
| Other medical specialties | Geriatrician | 27 |
| Other medical specialties | Hemostasis specialist | 30 |
| Other medical specialties | Occupational physician | 48 |
| Other medical specialties | Transfusion medicine specialist | 99 |
| Other medical specialties | Geneticist | 107 |
| Other medical specialties | Tuberculosis specialist | 186 |
| Other medical specialties | Hematologist | 298 |
| Other medical specialties | Infectious disease specialist | 330 |
| Other medical specialties | Nephrologist | 362 |
| Other medical specialties | Pulmonologist | 367 |
| Other medical specialties | Immunologist | 414 |
| Other medical specialties | Phlebologist | 450 |
| Other medical specialties | Allergist | 518 |
| Pediatrics | Neonatologist | 253 |
| Pediatrics | Pediatrician | 2389 |
| Psychiatry / Psychology | Sleep specialist | 58 |
| Psychiatry / Psychology | Sexologist | 69 |
| Psychiatry / Psychology | Neuropsychologist | 119 |
| Psychiatry / Psychology | Narcologist | 744 |
| Psychiatry / Psychology | Psychotherapist | 769 |
| Psychiatry / Psychology | Psychologist | 841 |
| Psychiatry / Psychology | Psychiatrist | 1892 |
| Surgery | Minimally invasive surgeon | 8 |
| Surgery | Oncoproctologist | 16 |
| Surgery | Hand surgeon | 18 |
| Surgery | Burn surgeon | 19 |
| Surgery | Laser surgeon | 73 |
| Surgery | Thoracic surgeon | 84 |
| Surgery | Cardiac surgeon | 263 |
| Surgery | Coloproctologist | 271 |
| Surgery | Maxillofacial surgeon | 298 |
| Surgery | Proctologist | 410 |
| Surgery | Neurosurgeon | 442 |
| Surgery | Plastic surgeon | 531 |
| Surgery | Vascular surgeon | 557 |
| Surgery | Trauma surgeon | 1472 |
| Surgery | Abdominal surgeon | 1614 |
| Surgery | Orthopedic surgeon | 1653 |
| Surgery | Surgeon | 3117 |
| Urology / Andrology | Andrologist | 534 |
| Urology / Andrology | Urologist | 1637 |

Supplementary Material Table SM2. Sex distribution of healthcare professionals across healthcare profession categories

| Healthcare profession category | Male, n (%) | Female, n (%) | Total, n |
| --- | --- | --- | --- |
| Anesthesia / Intensive care / Emergency medicine | 3,546 (60.29) | 2,336 (39.71) | 5,882 |
| Cardiology | 601 (24.82) | 1,820 (75.18) | 2,421 |
| Dentistry | 10,392 (46.21) | 12,098 (53.79) | 22,490 |
| Dermatology / Aesthetic medicine | 905 (13.76) | 5,672 (86.24) | 6,577 |
| ENT | 645 (33.16) | 1,300 (66.84) | 1,945 |
| Endocrinology / Diabetology | 175 (10.71) | 1,459 (89.29) | 1,634 |
| Gastroenterology | 654 (32.23) | 1,375 (67.77) | 2,029 |
| General practice / Internal medicine | 974 (20.53) | 3,770 (79.47) | 4,744 |
| Gynecology / Obstetrics | 1,153 (12.37) | 8,167 (87.63) | 9,320 |
| Imaging / Diagnostics | 1,706 (26.41) | 4,753 (73.59) | 6,459 |
| Musculoskeletal / Rheumatology / Rehabilitation | 741 (45.46) | 889 (54.54) | 1,630 |
| Neurology | 1,077 (31.78) | 2,312 (68.22) | 3,389 |
| Non-physician / Complementary medicine | 2,279 (46.11) | 2,664 (53.89) | 4,943 |
| Oncology | 996 (56.56) | 765 (43.44) | 1,761 |
| Ophthalmology | 487 (25.46) | 1,426 (74.54) | 1,913 |
| Other | 970 (29.85) | 2,280 (70.15) | 3,250 |
| Pediatrics | 311 (11.77) | 2,331 (88.23) | 2,642 |
| Psychiatry / Psychology | 1,895 (42.19) | 2,597 (57.81) | 4,492 |
| Surgery | 8,541 (78.75) | 2,305 (21.25) | 10,846 |
| Urology / Andrology | 1,837 (84.62) | 334 (15.38) | 2,171 |
| Overall | 39,885 (39.67) | 60,653 (60.33) | 100,538 |

Note: The overall total exceeds 64,939 because healthcare professionals could be assigned to multiple healthcare profession categories.

Supplementary Table SM3. Descriptive statistics of ratings according to sex across healthcare profession categories

| Healthcare profession category | n | Mean | SD | Median | IQR | Min | Max | n | Mean | SD | Median | IQR | Min | Max |
| --- | --- | --- | --- | --- | --- | --- | --- | --- | --- | --- | --- | --- | --- | --- |
|  | Male |  |  |  |  |  |  | Female |  |  |  |  |  |  |
| Anesthesia / Intensive care / Emergency medicine | 3546 | 8.536999 | 0.397668 | 8.6 | 0.8 | 6.4 | 9.8 | 2336 | 8.452997 | 0.4421182 | 8.6 | 0.6 | 3 | 9.6 |
| Cardiology | 601 | 8.81198 | 0.5731692 | 8.9 | 0.9 | 3 | 10 | 1820 | 8.713626 | 0.5324449 | 8.6 | 0.7 | 3 | 10 |
| Dentistry | 10392 | 8.547286 | 0.4548272 | 8.6 | 0.6 | 3 | 10 | 12098 | 8.508935 | 0.410423 | 8.6 | 0.5 | 3 | 10 |
| Dermatology / Aesthetic medicine | 905 | 8.890166 | 0.5968969 | 8.9 | 0.7 | 7.4 | 10 | 5672 | 8.730853 | 0.5493493 | 8.6 | 0.8 | 3 | 10 |
| ENT | 645 | 8.80124 | 0.5871542 | 8.7 | 0.9 | 3 | 10 | 1300 | 8.784846 | 0.5432428 | 8.7 | 0.8 | 7.6 | 10 |
| Endocrinology / Diabetology | 175 | 8.835429 | 0.5380913 | 8.8 | 0.9 | 8 | 10 | 1459 | 8.731254 | 0.5136376 | 8.6 | 0.7 | 6.3 | 10 |
| Gastroenterology | 654 | 8.823394 | 0.5340935 | 8.8 | 0.8 | 7 | 10 | 1375 | 8.8376 | 0.582662 | 8.8 | 0.8 | 2 | 10 |
| General practice / Internal medicine | 974 | 8.595483 | 0.5269511 | 8.6 | 0.8 | 3 | 10 | 3770 | 8.583952 | 0.4920916 | 8.6 | 0.8 | 3 | 10 |
| Gynecology / Obstetrics | 1153 | 8.828794 | 0.5181129 | 8.8 | 0.9 | 8 | 10 | 8167 | 8.848451 | 0.5367784 | 8.8 | 0.7 | 6.4 | 10 |
| Imaging / Diagnostics | 1706 | 8.674209 | 0.4870554 | 8.6 | 0.6 | 5.6 | 10 | 4753 | 8.677425 | 0.4876781 | 8.6 | 0.6 | 3 | 10 |
| Musculoskeletal / Rheumatology / Rehabilitation | 741 | 8.95776 | 0.6457636 | 8.9 | 1.1 | 8 | 10 | 889 | 8.794488 | 0.5730067 | 8.7 | 0.8 | 7.8 | 10 |
| Neurology | 1077 | 8.835376 | 0.5713922 | 8.8 | 0.9 | 6 | 10 | 2312 | 8.747016 | 0.5250206 | 8.6 | 0.7 | 5.8 | 10 |
| Non-physician / Complementary medicine | 2279 | 8.732646 | 0.6022606 | 8.8 | 0.9 | 3 | 10 | 2664 | 8.663964 | 0.5352187 | 8.6 | 0.7 | 3 | 10 |
| Oncology | 996 | 8.933534 | 0.538059 | 8.9 | 0.7 | 8 | 10 | 765 | 8.798431 | 0.5611531 | 8.7 | 0.9 | 6.9 | 10 |
| Ophthalmology | 487 | 8.828542 | 0.5325868 | 8.9 | 0.9 | 7.7 | 10 | 1426 | 8.753647 | 0.5193829 | 8.6 | 0.7 | 6 | 10 |
| Other | 970 | 8.925876 | 0.5780296 | 8.9 | 0.7 | 7.9 | 10 | 2280 | 8.784693 | 0.56094 | 8.6 | 0.8 | 6.6 | 10 |
| Pediatrics | 311 | 8.590032 | 0.4795801 | 8.6 | 0.9 | 7.9 | 10 | 2331 | 8.532046 | 0.4722861 | 8.6 | 0.8 | 3 | 10 |
| Psychiatry / Psychology | 1895 | 8.686227 | 0.5697544 | 8.6 | 0.7 | 3 | 10 | 2597 | 8.623566 | 0.5393091 | 8.6 | 0.7 | 4.4 | 10 |
| Surgery | 8541 | 8.79705 | 0.547458 | 8.7 | 0.8 | 6 | 10 | 2305 | 8.724729 | 0.5457102 | 8.6 | 0.6 | 6.9 | 10 |
| Urology / Andrology | 1837 | 8.953838 | 0.548818 | 8.9 | 0.7 | 7 | 10 | 334 | 8.842814 | 0.5409306 | 8.8 | 0.7 | 6.9 | 10 |
| Overall | 39885 | 8.710209 | 0.537342 | 8.6 | 0.6 | 3 | 10 | 60653 | 8.671647 | 0.5198039 | 8.6 | 0.7 | 2 | 10 |

Note: The overall total exceeds 64,939 because healthcare professionals could be assigned to multiple healthcare profession categories
